# STAT1*β* enhances STAT1 function by protecting STAT1*α* from degradation in esophageal squamous cell carcinoma

**DOI:** 10.1038/cddis.2017.481

**Published:** 2017-10-05

**Authors:** Ying Zhang, Yelong Chen, Hailong Yun, Zhaoyong Liu, Min Su, Raymond Lai

**Affiliations:** 1 Department of Pathology, Shantou University Medical College, Shantou, Guangdong Province, China; 2 Department of Orthopaedics, First Affiliated Hospital of Shantou University Medical College, 57 Changping Road, Shantou, Guangdong 515041, China; 3 Department of Laboratory Medicine and Pathology, University of Alberta, Edmonton, Alberta, Canada

## Abstract

STAT1, which carries tumor suppressor functions in several models, consists of two isoforms, namely STAT1*α* and STAT1*β*. The biological function and significance of STAT1*β* has never been examined in human cancer. We examined STAT1*β* function in esophageal squamous cell carcinoma (ESCC) by transfecting a STAT1*β* gene into various ESCC cell lines. The interaction between STAT1*α* and STAT1*β* was examined by using co-immunoprecipitation and confocal microscopy. The prognostic significance of STAT1*β* expression, detectable by immunohistochemistry and western blot, was evaluated in a large cohort of ESCC patients. Enforced expression of STAT1*β* induced and prolonged the expression and phosphorylation of STAT1*α* in ESCC cells, and these effects were amplified by gamma-interferon (IFN-*γ*). We also found that STAT1*β* interacts with STAT1*α* and decreases STAT1*α* degradation by the proteasome. Moreover, STAT1*β* substantially increased the DNA binding and transcription activity of STAT1. STAT1*β* also sensitized ESCC cells to chemotherapeutic agents, including cisplatin and 5-flurouracil. Using western blot and immunohistochemistry, we found that STAT1*β* was frequently decreased in esophageal cancer, as compared to their adjacent benign esophageal epithelial tissue. Loss of STAT1*β* significantly correlated with lymph node metastasis, invasion and shorter overall survival in ESCC patients. Therefore, STAT1*β* plays a key role in enhancing the tumor suppressor function of STAT1*α*, in ESCC, in a manner that can be amplified by IFN-*γ*.

Signal transducer and activator of transcription 1 (STAT1), a key mediator of interferon (IFN) signaling, regulates a variety of cellular activities, such as apoptosis, proliferation and differentiation.^1^ In response to extracellular stimuli, such as IFN-*γ*, activation of STAT1 is achieved by Janus kinase-mediated phosphorylation of its conserved tyrosine and serine residues, present in the C-terminal transactivation domain, to result in STAT1 dimerization, nuclear translocation, DNA binding and eventually modulation of expression of its target genes.^2^ In a number of models, STAT1 has been shown to possess tumor suppressor functions, and the evidence can be summarized as follows: (1) pro-apoptotic effects are largely mediated through STAT1 signaling,^2^ (2) constitutively active STAT1 can effectively induce apoptosis and inhibit cell growth,^3^ (3) STAT1 is frequently downregulated in various human cancers, including breast cancer, head and neck cancer, multiple myeloma and leukemia.^4, 5^ In our previous studies, we have reported that STAT1 is an important tumor suppressor in esophageal squamous cell carcinoma (ESCC), where loss of STAT1 contributes to the pathogenesis of these tumors and correlates with a worse clinical outcome.^6, 7^

STAT1*β*, a naturally spliced isoform of STAT1, lacks a 38-amino acid segment that includes the conserved STAT1^S727^ phosphorylation site and most of the C-terminal transactivation domain. STAT1*β* has not been extensively studied, although one report has described that STAT1*β* in human B-cells is transcriptionally inactive and exerts a dominant-negative effect on STAT1*α*, the full-length STAT1 isoform.^8^ Specifically, overexpression of STAT1*β* was found to inhibit the phosphorylation, DNA binding and transcriptional activity of STAT1 in human B-cells.^8^ However, in another study using B-cells, STAT1*β* was reported to induce cell death via a mechanism that is independent of p53 and STAT1*α*.^9^ In a more recently published paper, STAT1*β* was found to be transcriptionally active and capable of eliciting IFN-*γ*-dependent immunity against infection *in vivo*.^10^ Nonetheless, the biological function and clinical significance of STAT1*β* in human cancers has never been examined, and whether STAT1*β* possesses tumor suppressor activity is unknown.

Using ESCC cell lines as a model, we examined the biological and clinical significance of STAT1*β*. Our results support the concept that STAT1*β* enhances the expression and tumor suppressor function of STAT1*α*, and this effect can be amplified by IFN-*γ* stimulation. within support of this concept, loss of STAT1*β* in ESCC tumors correlates with a significantly worse clinical outcome.

## Results

### STAT1*β* increases expression and tyrosine phosphorylation of STAT1*α*

**Figure 1:**
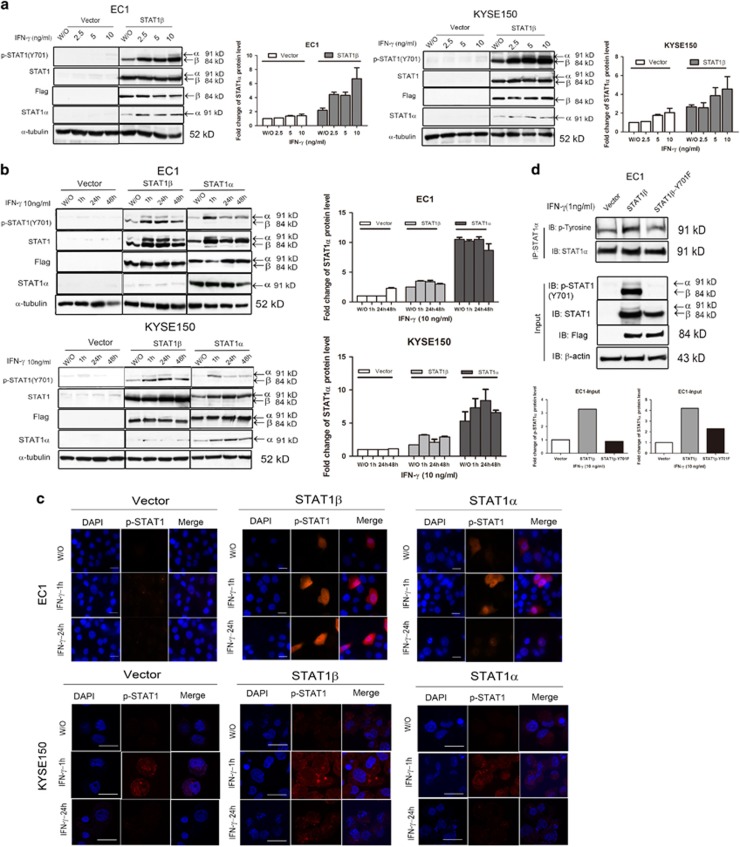
STAT1*β* increases and prolongs tyrosine 701 phosphorylation of STAT1*α*. (**a**) ESCC cell lines EC1 and KYSE150 were stimulated with IFN-*γ*, at the doses indicated, or left untreated (w/o) after empty vector or Flag-tagged STAT1*β* transfection. Total-protein extracts were used for detection of Tyr701-phosphorylated and total STAT1, flag and STAT1*α* by western blotting. (**b**) Both cell lines were stimulated with IFN-*γ* (10 ng/ml) for the time indicated, or left untreated (w/o) after empty vector or Flag-tagged *STAT1β* and *STAT1α* transfection. (**c**) EC1 and KYSE150 cells were stimulated with IFN-*γ* (10 ng/ml) for the indicated times after empty vector or Flag-tagged STAT1*β* and STAT1*α* transfection. Phospho-STAT1 was detected with an Alexa Fluor 568-conjugated secondary antibody (red). DAPI (1 *μ*g/ml) was used for nuclear staining (blue). Fluorescence signals were analyzed with a Zeiss LSM 710 confocal microscope (scale bar 5 *μ*m). (**d**) Tyrosine phosphorylation of STAT1*α* in EC1 cells was detected by immunoprecipitation and western blotting, after empty vector or Flag-tagged *STAT1β* or *STAT1β*^*Y701F*^ plasmid transfection, upon IFN-*γ* stimulation. Data are representative of three independent experiments

Both ESCC cell lines (EC1 and KYSE150) had no detectable expression of STAT1 or p-STAT1^Y701^ after the transfection of an empty vector (Figure 1a, *left*). At 12 h after the addition of varying doses of IFN-*γ*, there was also no appreciable change in the expression of STAT1 and p-STAT1^Y701^. In contrast, cells transfected with *STAT1β* showed detectable STAT1*α* expression (Figure 1a, *right*). At 12 h after the addition of various amounts of IFN-*γ*, the total STAT1*α* as well as p-STAT1^Y701^ (containing both STAT1*α* and STAT1*β*) levels increased in a dose-dependent manner. Time course experiments revealed similar results. As shown in the middle panel of Figure 1b, following *STAT1β* transfection, IFN-*γ* addition resulted in a rapid and dramatic increase in STAT1*α* as well as p-STAT1^Y701^. Importantly, the enhancement of p-STAT1*α* by STAT1*β* was almost as potent and sustained as by *STAT1α* transfection.

To further substantiate our finding that STAT1*β* increases the expression and phosphorylation of STAT1*α*, we performed confocal microscopy using EC1 and KYSE150. No nuclear p-STAT1^Y701^ was detectable at 1 h or 24 h after IFN-*γ* stimulation (Figure 1c). In contrast, in *STAT1β*-transfected cells, a strong nuclear p-STAT1^Y701^ signal was detectable at 1 h, after IFN-*γ* addition, then declined by 24 h. Similar findings were found following *STAT1α* transfection, although the p-STAT1^Y701^ signal was slightly more intense than that resulting from *STAT1β* transfection. These results correlated well with the western blot results illustrated in Figure 1b.

We then performed immunoprecipitation. Tyrosine phosphorylation of STAT1*α* in EC1 cells was increased in the presence of STAT1*β*, but not the empty vector (Figure 1d). Importantly, tyrosine phosphorylation of STAT1*α* was largely abrogated when the *STAT1β*^*Y701F*^ mutant, instead of wild-type *STAT1β*, was used for transfection. This finding highlights the importance of the activation/phosphorylation of STAT1*β* in potentiating the expression and phosphorylation of STAT1*α*.

### STAT1*β* interacts with STAT1*α* and protects STAT1*α* from proteasome degradation

**Figure 2:**
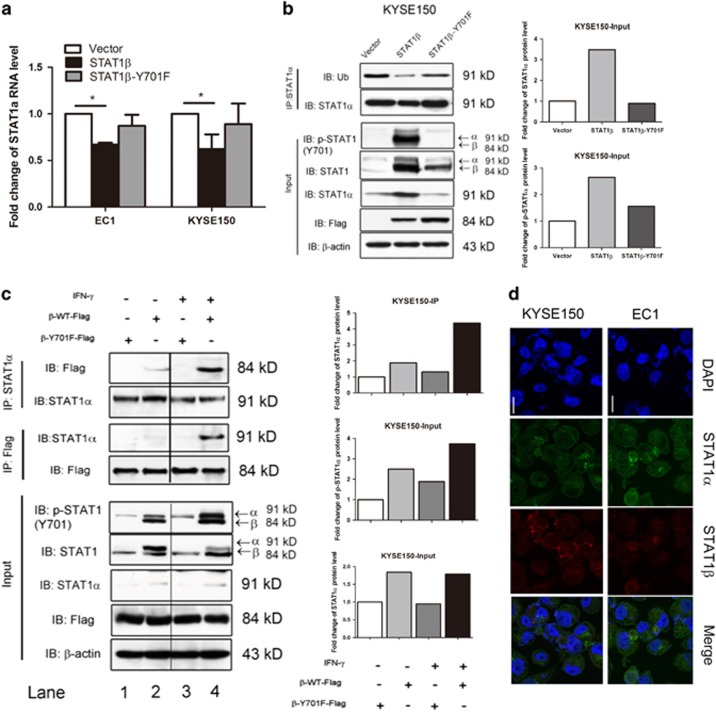
STAT1*β* interacts with STAT1*α* to protect STAT1*α* from proteasome degradation. (**a**) STAT1*α* mRNA expression was detected by real-time PCR after transfection of empty vector or Flag-tagged *STAT1β* or *STAT1β*^*Y701F*^ plasmids. Values were normalized to GAPDH and calculated relative to empty vector-transfected cells. Mean values and standard errors (SE) from at least three independent experiments are shown. (**b**) Immunoprecipitation–immunoblotting analysis was performed for STAT1*α* and ubiquitination in EC1 cells transfected with empty vector or Flag-tagged *STAT1β* or *STAT1β*^*Y701F*^ plasmids. (**c**) The interaction of STAT1*α* and STAT1*β* was investigated by immunoprecipitation and western blot analysis in EC1 cells with or without IFN-*γ* stimulation. Co-immunoprecipitaion was carried out with control IgG and anti-Flag or anti-STAT1*α* antibodies as indicated. Immunoprecipitated proteins were analyzed by western blot with anti-STAT1*α* and anti-Flag, respectively. (**d**) Co-localization of STAT1*α* and STAT1*β in vivo*. EC1 and KYSE150 cells were placed on coverslips and stained with the indicated antibodies (scale bar 5 *μ*m)

To investigate the mechanisms by which STAT1*β* enhances STAT1*α* expression and phosphorylation, we asked if STAT1*β* increased the expression of *STAT1α* mRNA. By quantitative RT-PCR, we found a significant decrease in STAT1*α* mRNA after *STAT1β* transfection in both ESCC cell lines, whereas transfection of the *STAT1β*^*Y701F*^ mutant did not have any appreciable effect (Figure 2a). Since the relatively low STAT1 expression in ESCC can be attributed to its degradation via the ubiquitin-proteasome pathway (manuscript submitted), we tested if this pathway is also involved in the upregulation of STAT1*α* mediated by STAT1*β*. In keeping this hypothesis, we performed immunoprecipitation using an antibody against STAT1*α*. As shown in Figure 2b, we found that transfection of *STAT1β* almost completely abrogated STAT1*α* ubiquitination. Consistent with our previous data, the Y701F mutation of *STAT1β* lacked this effect.

To further investigate the relationship between STAT1*α* and STAT1*β*, we performed co-immunoprecipitation and western blot experiments. By co-immunoprecipitation experiments (Figure 2c), in the absence of IFN-*γ* (lanes 1 and 2), we found evidence of physical binding between Flag-tagged STAT1*β* and STAT1*α* in KYSE150 cells. This effect was amplified when IFN-*γ* was added (Figure 2c, lanes 3 and 4). The physical interaction between these two STAT1 isoforms was further supported by our confocal microscopy results (Figure 2d). In contrast, there was no detectable physical binding between Flag-tagged STAT1*β*^Y701F^. Taken together, our results suggest a model in which STAT1*β* protects STAT1*α* from proteosomal degradation, thus increasing the total protein level of STAT1*α*.

### STAT1*β* enhances DNA binding and transcription activity of STAT1*α*

**Figure 3:**
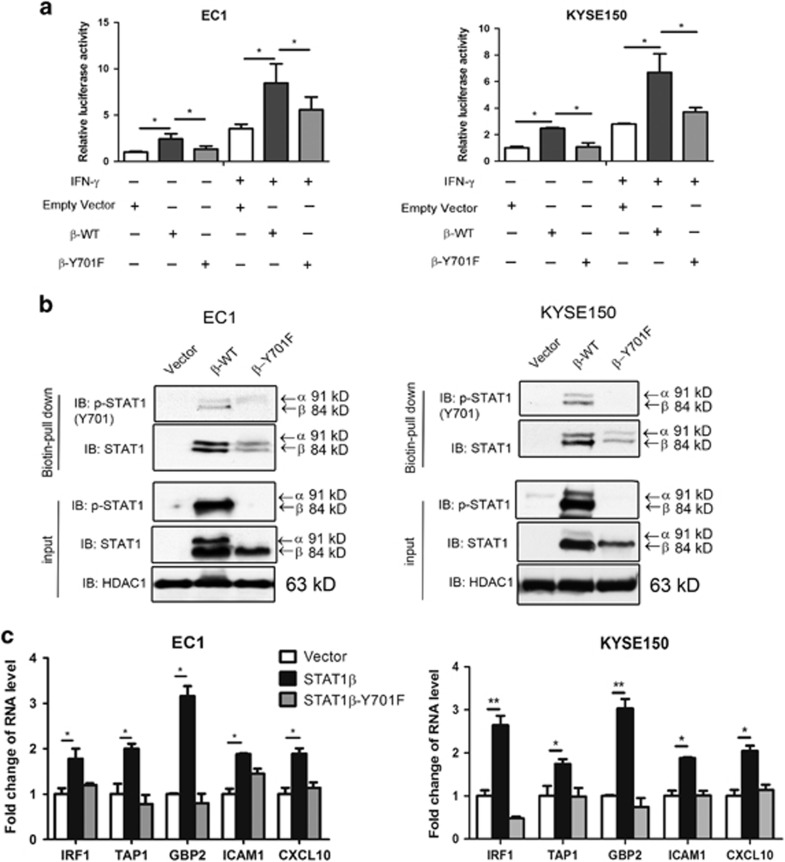
STAT1*β* enhances transcription activity and DNA binding of STAT1. (**a**) EC1 and KYSE150 cell lines were transfected with either empty vector, or Flag-tagged *STAT1β* or *STAT1β*^*Y701F*^ plasmids, then stimulated with IFN-*γ*. After 48 h, the transcription activity of STAT1 was detected by a dual-luciferase reporter assay. (**b**) After transfection with *STAT1β* or *STAT1β*^*Y701F*^ plasmids, the DNA binding ability of STAT1 was detected, in both cell lines, with a STAT1 biotin-probe followed by western blotting. (**c**) Total RNA was extracted and used for RT-qPCR analysis for the genes indicated. *GAPDH* was used for normalization, and expression levels were calculated relative to empty vector-transfected cells. Triplicate experiments were performed and results from a representative experiment are shown. *P< 0.05; **P<0.01

Using a STAT1 luciferase reporter, we assessed the effect of STAT1*β* on the transcriptional activity of STAT1 in ESCC cell lines. As shown in Figure 3a, *STAT1β* transfection significantly increased the transcriptional activity of STAT1, as compared to that of empty vector (*P*<0.05). Again, transfection of the *STAT1β*^*Y701F*^ mutant yielded only a minimal increase, as compared to empty vector (*P*=0.71). Similar observations were observed when transfected cells were stimulated with IFN-*γ*, although the effects were more profound.

To test whether the increased transcriptional activity of STAT1 mediated by *STAT1β* transfection was caused by an increase in STAT1-DNA binding, we performed pull-down experiments using a biotinylated probe containing the STAT1 DNA-binding consensus sequence. As shown in Figure 3b, upon IFN-*γ* stimulation, STAT1-DNA was markedly enhanced by *STAT1β* transfection, as compared to that of empty vector or *STAT1β*^*Y701F*^.

To confirm the effect of STAT1*β* on gene transcription, we performed quantitative RT-PCR. ESCC cells were transiently transfected with either empty vector, wild-type *STAT1β* or *STAT1β*^*Y701F*^, then the mRNA expression levels of several known STAT1 downstream targets (including *IRF1, TAP1, CXCL10, GBP2* and *ICAM10*) were analyzed.^11^ Compared to transfection with empty vector, *STAT1β* transfection at 24 h significantly increased the mRNA expression of all five target genes examined (Figure 3c). As above, enforced expression of the STAT1^Y701F^ mutant did not appreciably increase the expression of these five genes.

### STAT1*β* enhances the tumor suppressor function of STAT1*α*

**Figure 4:**
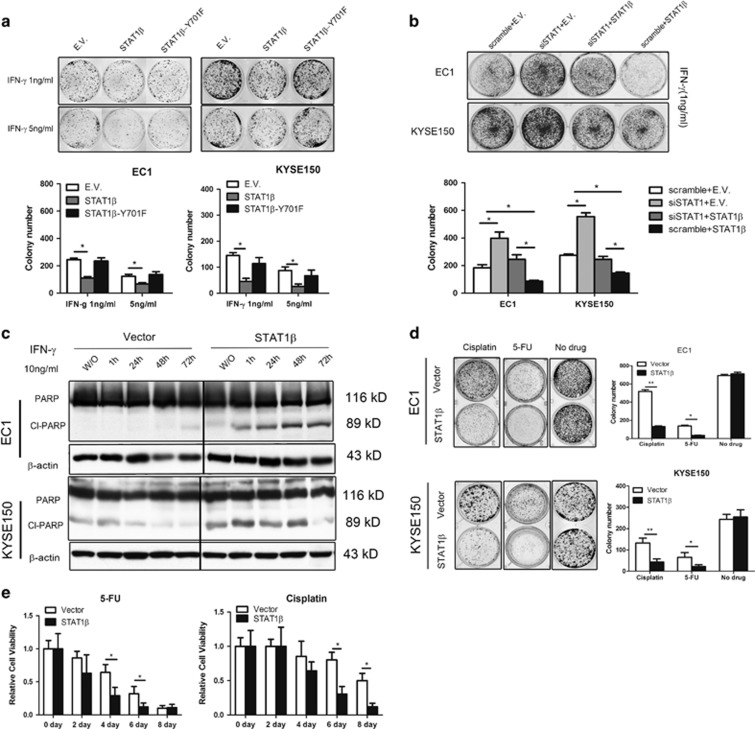
Biological functions of STAT1*β* in ESCC. (**a**) Tumorigenicity, assessed by colony formation assay, was detected in EC1 and KYSE150 cells, transfected with *STAT1β*, *STAT1β*^*Y701F*^ or empty vector, at different doses of IFN-*γ* stimulation after 10 days. (**b**) In both cell lines, colony formation was performed in the cells with siRNA knockdown of *STAT1* and transfection of *STAT1β*. (**c**) Using western blot analysis, cleaved and total PARP expression was detected in EC1 and KYSE150 cells transfected with *STAT1β* or empty vector. (**d**) The chemosensitivity to 5-FU and cisplatin of ESCC cells was assessed by colony formation after transfection of *STAT1β* for 10 days. (**e**) Cell growth, as assessed by MTS assay, was detected after *STAT1β* transfection in EC1 and KYSE150 cells after a 4-day exposure to IFN-*γ*. Triplicate experiments were performed and results from a representative experiment are shown (*P<0.05)

To evaluate the biological effect of STAT1*β* in ESCC cells, we performed colony formation assays using EC1 and KYSE150 cells. Transfection of *STAT1β* significantly decreased soft-agar colony formation of both cell lines (*P*<0.05), whereas STAT1*β*^Y701F^ did not show similar tumor suppressor effects (Figure 4a). To support the functional role of STAT1*β*, we knocked down STAT1 and then measured soft agar colony formation in the presence of IFN-*γ* (1 ng/ml) (Figure 4b). Upon STAT1 siRNA knockdown, both EC1 and KYSE150 cells showed a significant increase in the number of colonies. However, enforced STAT1*β* expression significantly attenuated the biological effect of STAT1 siRNA knockdown (lane 3). Furthermore, enforced expression of STAT1*β* in the absence of STAT1 siRNA knockdown (lane 4) brought the number of colonies to the lowest level in both cell lines.

Western blotting revealed that transfection of *STAT1β* led to the expression of cleaved poly-ADP ribose polymerase (PARP) in ESCC cells stimulated with IFN-*γ* (Figure 4c). Also, *STAT1β* transfection synergized with 5-fluorouracil (5-FU) and cisplatin in decreasing colony formation (Figure 4d), as well as the number of viable cells in an MTS assay (Figure 4e).

### Expression of STAT1*β* in ESCC tumors and its prognostic significance

**Figure 5:**
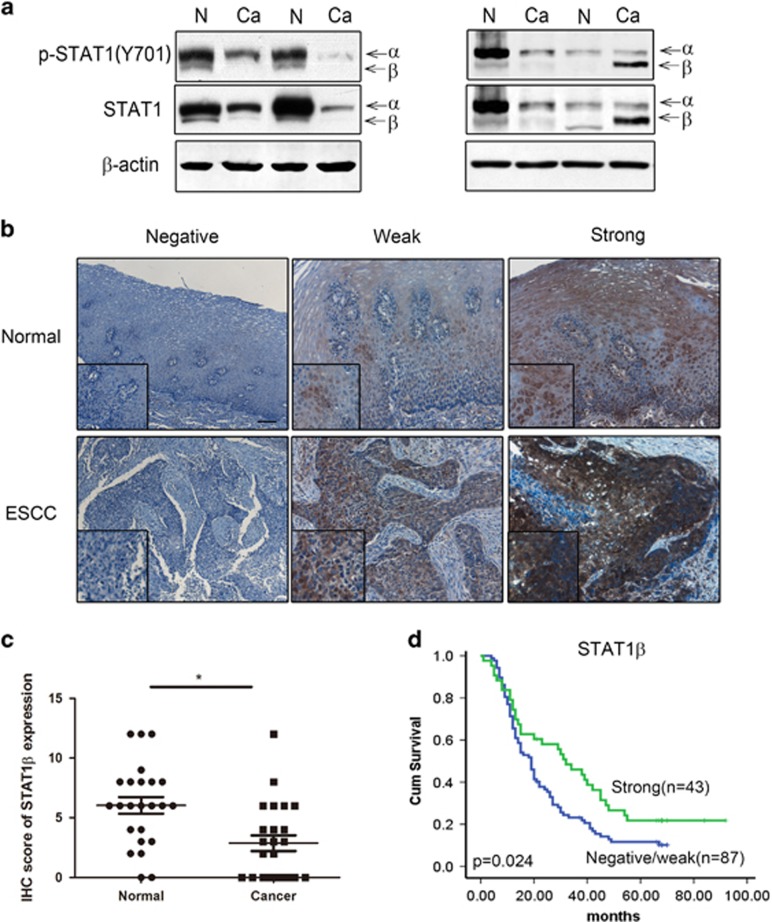
Expression of STAT1*β* in ESCC patient samples. (**a**). STAT1*β* expression in ESCC tumors was examined by western blot. Compared to benign esophageal tissue harvested at the surgical margins in the same specimens (labeled N), cancerous tissue (labeled Ca) often expressed a lower level of STAT1*β* (e.g. cases 1–3). A small subset of tumors (e.g. case 4) had high levels of STAT1*β*. (**b**) Immunohistochemistry of formalin-fixed, paraffin-embedded tissues showed variable levels of predominantly cytoplasmic STAT1*β* were detectable in esophageal epithelial and ESCC tissues. Based on the staining intensity, normal epithelia and tumors in our cohort were categorized into STAT1-negative, STAT1-weak or STAT1-strong (IHC stain, scale bar, 20 *μ*m). (**c**) The immunohistochemistry scores of STAT1*β* showed that expression of STAT1*β* is higher in normal tissues compared to cancer tissues. (**d**) By Kaplan–Meier analysis, a significant correlation between overall survival and the expression level of STAT1*β* was found between patients with STAT1*β*-strong and STAT1*β*-weak/negative staining (*P<0.05)

By western blot, we detected the expression of p-STAT1^Y701^ and total STAT1 in 12 ESCC tumors and their case-matched, adjacent normal tissues. As illustrated in Figure 5a, all 12 cases showed low levels of expression of the STAT1*α* isoform and p-STAT1*α*^Y701^ (the upper bands). Most (8/12, 66.6%) tumors showed lower expression of p-STAT1*β*^Y701^ and STAT1*β* (the lower bands); only (16.7%) cases showed a slight increase and another 2 (16.7%) cases showed no appreciable difference.

We then performed immunohistochemistry (IHC) using 33 ESCC tumors and the case-matched, adjacent normal tissues. As illustrated in Figures 5b and c, STAT1*β* immunoreactivity was found in 29 (87.9%) cases of benign esophageal epithelial tissues, but only 19 (57.6%) of ESCC tumors. Using our IHC scoring method, we found that ESCC tumors had lower STAT1*β* expression than the benign epithelia, in keeping with the concept that STAT1*β* is a tumor suppressor and its expression is frequently lost during the carcinogenesis of ESCC.

In view of its tumor suppressor function, the prognostic significance of STAT1*β* was evaluated in a cohort of 201 ESCC tumors. We found that STAT1*β* was undetectable in 94 (46.8%) cases, weakly expressed in 47 (23.3%), and strongly expressed in 60 (29.9%) cases. By comparing the STAT1*β* staining intensity (negative/weak or strong) and total STAT1 staining obtained from one of our previous studies,^6^ we found a positive correlation (*R*=0.765, *P*<0.0001, illustrated in Table 1). The correlation of STAT1*β* and various clinical parameters was also assessed (Table 2), Negative/weak STAT1*β* staining significantly correlated with depth of invasion (*P*<0.001), lymph node metastasis (*P*=0.045) and a high clinical stage (*P*=0.026). Clinical follow-up data were available in 130 of 201 cases included in this study (median follow-up, 21.5 months, and range 5–92 months). The prognostic value of STAT1*β* was analyzed by Kaplan–Meier survival analysis and the cut-point was determined by X-tile software. As shown in Figure 5d, patients with expression of total STAT1*β*^strong^ (*n*=43) had a significantly better clinical outcome compared to the STAT1*β*^negative/weak^ (*n*=87, *P*=0.025). STAT1^strong^/STAT1*β*^strong^ patients (*n*=27) had longer survival than both the STAT1^strong^/STAT1*β*^negative/weak^ patients (*n*=12) and STAT1^negative/weak^/STAT1*β*^negative/weak^ patients (*n*=35) (Supplementary Figure 1).

## Discussion

We previously demonstrated that STAT1 is a tumor suppressor in ESCC.^6, 7^ Similar to STAT3 and STAT4, STAT1 has two isoforms, namely STAT1*α* and STAT1*β*. STAT1*α* is the full-length isoform and is considered to be the transcriptionally active form of STAT1, which is also known to form complexes with other transcription factors to modulate gene expression in normal cells.^10^ STAT1*α* has been reported to mediate various cellular activities, including inhibition of cell growth, cell cycle arrest and apoptosis.^2^ In comparison, the function of STAT1*β* has not been extensively studied. STAT1*β* is the truncated form of STAT1 and lacks a 38-amino acid segment that includes most of the transactivation domain and the functionally important serine 727.^12^ Corresponding to its truncated structure, it has been shown that STAT1*β* can bind to the promoter of genes such as IRF1, LMP2 and TAP1, but is transcriptionally inactive.^13, 14^

As mentioned previously, STAT1*β* is believed to exert dominant negative effect on STAT1*α*, due to its lack of the transactivation domain and regulatory serine 727 phosphorylation site. This concept came from a study of human B cells, in which enforced expression of STAT1*β* was found to inhibit STAT1*α* activation by decreasing the tyrosine 701 phosphorylation, DNA binding and transcriptional activity of STAT1*α*, as well as protecting the cells from fludarabine-induced apoptosis.^8^ In keeping with this concept, infection with *Mycobacterium tuberculosis* and *Leishmania Mexicana* has been found to increase the expression and phosphorylation of STAT1*β*, leading to an inhibition of STAT1 signaling.^15, 16^ In another study, there is evidence that the ratio of STAT1*α*/STAT1*β* may affect the sensitivity of cells to viral infection.^17^ The mechanism of how STAT1*β* inhibits STAT1*α* is incompletely understood. One possible explanation is that STAT1*β* competes with STAT1*α* for the same DNA binding sites, but is incapable of activating the gene expression.^8, 15, 16^ Another possible explanation is that STAT1*β* may compete with STAT1*α* for the same receptor sites, thus interfering with STAT1*α* activation.

Results from a more recent study challenge the view that STAT1*β* is simply an inhibitor of STAT1*α*. Specifically, STAT1*β* has been found to induce death in human B cells, independently of p53 or STAT1*α*.^9^ In a recently published paper, it was reported that STAT1*β* is transcriptionally activated in response to IFN-*γ*, and IFN-*γ*-induced tyrosine phosphorylation and promoter binding of STAT1 is prolonged in the absence of STAT1*α*.^10^ In the same study, it also was reported that STAT1*β* can induce the expression of many gene targets of STAT1 upon IFN-*γ* stimulation.^10^

Our findings from this current study also challenge the view that STAT1*β* is an inhibitor of STAT1*α*. We find that STAT1*β* can increase the expression of STAT1*α* and potentiate its activation/phosphorylation mediated by IFN-*γ*. Indeed, we found that STAT1*β* substantially enhances the DNA binding and transcription activity of STAT1*α* in ESCC cells and STAT1*β* can modulate the expression of known STAT1 gene targets, including IRF1, TAP1 and GPB2. Probably through effects on STAT1*α*, STAT1*β* exerts tumor suppressor effects in ESCC. It is clear that our findings, and others, contradict previous studies regarding STAT1*β*, as discussed above. While the explanations for this discrepancy are not clear, we consider that cell-type specificity is likely a contributing factor. Researchers also demonstrated prolonged phosphorylation of STAT1 after STAT1*β* transfection, and that this effect is related to a reduction of SOCS1,^10^ which is a negative regulator of the JAK-STAT1 pathway.^18^ However, in our study, we did not find a change in SOCS1 expression by ESCC cells after STAT1*β* transfection.

**Figure 6:**
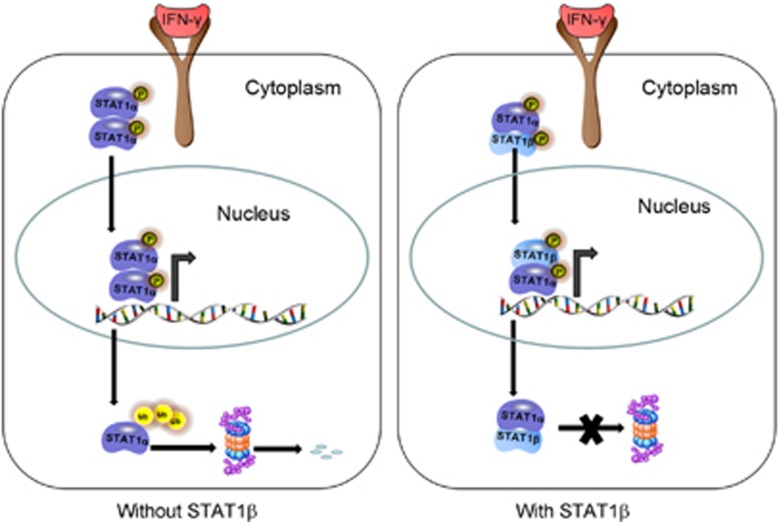
Schematic diagram showing STAT1*β* interacts with STAT1*α*. Upon IFN-*γ* stimulation, STAT1 can form three different dimers: *α*:*α* and *β*:*β* homodimers and *α*:*β* heterdimers. Dimers can be phosphorylated followed by translocation into the nucleus. Once STAT1 is released from the target gene promoter, it returns to the cytoplasm and is degraded by the proteasome. However, the STAT1*β* can bind to STAT1*α* to protect STAT1*α* from degradation, resulting in prolongation of the half-life of STAT1*α* and a concomitant increase in transcription activity

Our findings also lead us to believe that the key mechanisms underlying the effects of STAT1*β* on STAT1*α* are related to its binding to STAT1*α*, thereby protecting it from being degraded via the proteasomal pathway. In support of the concept that STAT1*β* potentiates the tumor suppressor effect of STAT1*α*, we find that loss of STAT1*β* expression significantly correlates with a worse clinical outcome in a large cohort of ESCC patients. Our hypothetical model is summarized in Figure 6.

Similar to STAT1*β*, both STAT3*β* and STAT4*β* have been reported to be transcriptionally active and able to carry their unique functions.^19^ There are at least two mechanisms underlying the transcription activity and biological function of STAT1*β*. One mechanism is that the beta isoforms of STATs can interact with other transcription factors that provide a C-terminal transactivation domain. For example, STAT3*β* can cooperate with c-Jun to activate the *α*-macroglobulin promoter.^20^ Thus, gene transfection of *STAT3β* results in increasing STAT3*α* transcription activity.^21, 22^ Another mechanism is that STAT1*β* still retains the most important functional domain. For example, the abbreviated transactivation domain contains the tyrosine 701 phosphorylation site that is essential for various STAT1 functions. The N-terminal domain can interact with CBP, with low affinity, to exert low-level transcriptional activation,^22, 23^ and the SH2 domains can participate in dimerization of STAT1 to bind to receptors.^24^ Interaction of the coiled-coil domain and the DNA binding domain can form antiparallel STAT1 dimers.^25^ Although STAT1*β* lacks the serine 727 phosphorylation site, it is likely that STAT1*β* can mediate some degree of transcription activity of STAT1, since serine 727 has been reported to be necessary for the maximal activation of STAT1.^22^

The biological function and clinical significance of STAT1*β* has yet to be reported in human cancers. Moreover, no paper has described the expression of STAT1*β* in human normal or cancer tissues Therefore, in our paper, we firstly used ESCC cells as a model to determine the biological function of STAT1*β*. We show cells transfected with *STAT1β* are more sensitive to IFN-*γ* and anti-tumor drugs, such as 5-FU and cisplatin. These findings suggest that, in ESCC, STAT1*β* also exerts a tumor suppressor function. Analysis of ESCC patient samples also supports our hypothesis. In a previous paper, we found that expression of STAT1*β* is significantly higher in immortalized esophageal cell lines than in ESCC cell lines, indicating that STAT1*β* is downregulated in ESCC tumorigenesis.^6^ We further show that STAT1*β* is downregulated in ESCC tissues compared to case-matched normal esophageal epithelia, which is similar to our previous findings on STAT1 in ESCC. STAT1*β* expression is strongly related to the total expression of STAT1, which supports our hypothesis that STAT1*β* may stabilize STAT1*α* to modulate and increase total STAT1 expression. Another key finding of our study is that low STAT1*β* expression correlates with greater tumor invasion and lymph node metastasis, and worse clinical outcome.

In conclusion, we investigated the biological function and clinical significance of STAT1*β* in human cancer. In ESCC cells, STAT1*β* enhances the tumor suppressor function of STAT1 by increasing the expression and activation of STAT1*α*. The *in vivo* results also indicate that STAT1*β* is downregulated in the ESCC carcinogenesis, and correlates with worse clinical outcome. Our study challenges the concept that STAT1*β* is simply a dominant negative regulator of STAT1 and provides a new therapeutic target for treating ESCC patients.

## Materials and methods

### Cells and patient samples

Human ESCC cell lines, EC1 and KYSE 150, were used in this study. Cells were maintained in RPMI 1640 (KYSE150), or Dulbecco’s modified Eagle’s medium supplemented (EC1) with 10% fetal bovine serum, and 1 × antibiotic mixture (Invitrogen, Carlsbad, CA, USA). All cells were cultured at 37 °C in a humidified incubator containing 5% CO_2_.

We randomly collected 201 consecutive ESCC samples at the Shantou Tumor Hospital between 2005 and 2012. All patients underwent potentially curative surgery without preoperative chemotherapy or radiotherapy. In this cohort, 150 were men and 51 were women; the range of ages was 36–81 years, with a median of 57 years. Follow-up data were available for 130 patients; most (113, 86.9%) died during the follow-up period (median survival, 21.5 months). Of the 201 ESCC tumors, 33 case-matched normal esophageal tissues adjacent to the tumors were included in the study. Written informed consents were obtained from the patients, and the study was reviewed and approved by the institutional ethics committee of Shantou University Medical College.

### Western blot analysis

A tissue lyser (Qiagen, Valencia, CA, USA) was used to prepare frozen tumor samples for western blot analysis. Cell lines and tumor samples were lysed in RIPA buffer containing protease inhibitor cocktail and Set II phosphatase inhibitor cocktail. Total tissue extracts were stored on ice for 20 min and then centrifuged at 13 000 r.p.m. at 4 °C for 15 min. Proteins from the supernatant were measured using a bicinchoninic acid assay (Thermo scientific, Rockford, IL, USA). Equal amounts of cell lysate were separated by 10% sodium dodecyl sulfate (SDS)–polyacrylamide gel electrophoresis and evaluated by western blot analysis as described previously.^26^ Antibodies reactive with human *β*-actin (1:1000), STAT1 (1:1000), phospho-Tyr 701 STAT1 (or p-STAT1) (1:1000), Flag (1:1000), and PARP (1:1000) were purchased from Cell Signalling (Danvers, MA, USA). Anti-STAT1*α* (1:1000) was purchased from Santa Cruz (Dallas, TX, USA), and anti-STAT1*β* (1:200) was purchased from SignalChem (Richmond, BC, Canada).

### Cell growth assay

The ESCC cell lines were transfected with STAT1*β*, mutant STAT1*β*^Y701A^ or empty vector. Then, 1 × 10^4^ transfected cells were grown in each well of a 96-well microplate and treated with anti-tumor drug, either cisplatin or 5-flurouracil (5-FU), for 0–8 days. At different times, the cells were incubated with 10 *μ*l MTS reagent (Invitrogen) for 1 h, and the increase in absorbance at 490 nm relative to the blank well control was measured using a microplate spectrophotometer.

### Plasmid constructs and gene transfection

Plasmids including Flag-STAT1*β* and STAT1*α* were purchased from Addgene (Cambridge, MA, USA). Mutant STAT1*β*^Y701F^ plasmid was engineered in our laboratory. For each experiment, 1 × 10^6^ ESCC cells were transiently transfected with 10 *μ*g of STAT1-expressing plasmid or empty vector, in six-well plates, using the lipofectamine 2000 reagent (Invitrogen) as per the manufacturer’s suggested protocol.

### Co-immunoprecipitation of STAT1*α* and STAT1*β*

To detect the interaction between STAT1*α* and STAT1*β*, whole-cell extracts were prepared by lysing the cells in an immunoprecipitation buffer. A total of 2 *μ*g of anti-STAT1*α* monoclonal antibody (Santa Cruz Biotechnology, Dallas, TX, USA) was added to 500 *μ*g of protein lysate isolated in cell lysis buffer (Sigma Aldrich, St Louis, MD, USA) and the samples were rotated overnight at 4 ^o^C. Subsequently, 30 *μ*l of protein G Plus/A beads (EMD Millipore, Billerica, MA, USA) was added to the samples and rocked overnight at 4 ^o^C. The beads were then washed three times with cold phosphate-buffered saline (PBS), followed by a final wash using cold cell lysis buffer or RIPA buffer. Western blot analysis was then performed using standard techniques as previously described.^26^

### Immunofluorescence and confocal microscopy

Cells were grown on cover slips coated with poly-L-lysine (Sigma Aldrich) in a six-well plate and fixed with 3% paraformaldehyde in PBS (pH 7.4). Cells were rinsed three times with PBS, permeabilized with Triton X-100, washed again with PBS, and incubated with 200 *μ*l of anti-p-STAT1 antibody (1:50, Sigma Aldrich) overnight at room temperature in a humidified chamber. The cover slips were rinsed three times in PBS and incubated with secondary antibody conjugated with Alexa Fluor 488 or 568 (Invitrogen) at a 1:250 dilution for 1 h at room temperature. After three rinses in PBS, the coverslips were mounted on a slide using mounting media (Dako, Mississauga, ON, Canada). Cells were visualized with a Zeiss LSM 710 confocal microscope at the Core Cell Imaging Facility, Cross Cancer Institute (Edmonton, AB, Canada).

### Colony formation assay

After STAT1*β* or empty vector transfection, cells were inoculated in six-well plates, at a density of 500 cells/well and incubated for 10 days at 37 °C. The cells were fixed with 4% buffered formalin for 15 min and then stained with 1% crystal violet (Sigma Aldrich) for 30 min. The plates were gently washed with PBS and dried before microscopic evaluation. Cell clusters with >30 cells were considered as a colony.

### Luciferase activity assay

STAT1 transcription activity was measured with a STAT1 luciferase reporter. Luciferase activity was measured with the Dual-Luciferase reporter assay system (Thermo Scientific) according to the manufacturer’s instructions as described. Data were normalized for transfection efficiency by division of firefly luciferase activity with that of Renilla luciferase.

### Subcellular fractionation and DNA binding assays

Nuclear and cytoplasmic protein of ESCC cells was extracted using an NE-PER protein extraction kit (Thermo Scientific) according to the manufacturer’s instructions. For western blot analysis, tubulin and histone deacetylase 1 (HDAC1) were used for the cytoplasmic and nuclear loading control, respectively.

Oligonucleotide pull-down assays were performed with an annealed nucleotide comprising the STAT1 consensus site (5′-CATGTTATGCATATTCCTGTAAGTG-3′) with a 5-biotin label. Nuclear extracts (50–100 *μ*g) were incubated for 1 h at 4 °C with 1 *μ*g oligonucleotide in binding buffer, then Sepharose–streptavidin (50 *μ*l; Sigma) was added for 2 h at 4 °C. After three washes in PBS, the complexes were suspended in SDS sample buffer and processed for western blotting, as described above, and probed with anti-STAT1 and anti-pSTAT1 antibodies (Cell Signalling).

### Quantitative RT-PCR

Using an RNeasy Mini Kit (Qiagen), total cellular RNA was extracted from cells following the manufacturer’s protocol. Primer sequences were: human interferon regulatory factor 1 (IRF1) forward: 5′-ATGCCCATCACTCGGATGC-3′, reverse: 5′-CCCTGCTTTGTATCGGCCTG-3′ human chemokine (C-X-C motif) ligand 10 (CXCL10) forward: 5′-GTGGCATTCAAGGAGTACCTC-3′, reverse: 5′-TGATGGCCTTCGATTCTGGATT-3′ STAT1*α* forward: 5′-CCAATGGAACTTGATGGCCC-3′, reverse: 5′-CAGAGCCCACTATCCGAGAC-3′ guanylate binding protein 2 (GBP2) forward: 5′-CTATCTGCAATTACGCAGCCT-3′, reverse: 5′-TGTTCTGGCTTCTTGGGATGA-3′ intercellular adhesion molecule 10 (ICAM10) forward: 5′-ATGCCCAGACATCTGTGTCC-3′, reverse: 5′-GGGGTCTCTATGCCCAACAA-3′ transporter associated with antigen processing 1 (TAP1) forward: 5′-GCAAGACGACTTACTCTGGGT-3′, reverse: 5′-GGATCTGACACCACTGGACC-3′.

### Immunohistochemistry

Formalin-fixed, paraffin-embedded ESCC tumors were used for this study. All cases were retrieved from the file at the Department of Pathology, Shantou University Medical College. The diagnosis of these cases was based on criteria established by the World Health Organization classification scheme. Immunohistochemistry to detect STAT1 expression was performed using a method similar to that described previously.^11^ Briefly, formalin-fixed, paraffin-embedded tissue sections of 4 *μ*m thickness were deparaffinized in xylene and hydrated in graded ethanol (100–50%). Antigen retrieval was performed by immersing tissue in citrate buffer (pH 6.0), microwaving in a pressure cooker for 20 min, and leaving to cool for 20 min at room temperature. After antigen retrieval, tissue sections were incubated with 10% hydrogen peroxide (H_2_O_2_) and methanol for 10 min to block endogenous peroxidase activity, followed by washing in running tap water for 5 min. Subsequently, the sections were incubated for 20 min in antibody diluent (Dako), followed by incubation overnight at 4 °C with a rabbit polyclonal antibody reactive with anti-STAT1*β* (1:200 dilution, Cellchemo, MA, USA). Immunostaining was visualized with a labeled streptavidin-biotin method (20 min in biotinylated link and 20 min in streptavidin-conjugated horse radish peroxidase, both from Dako) using (3, 3-diaminobenzidine/H_2_O_2_) DAB as a chromogen (Dako). Hematoxylin was used as a counter stain. Following staining, sections were dehydrated in graded ethanol (50–100%), followed by xylene incubation. Coverslips were applied using Permount solution (Fisher Scientific, Pittsburgh, PA, USA).

Staining was independently evaluated by two pathologists who were blinded to the clinical data. The percentages of positively stained cells were assigned the following scores: 0 (<5% positive), 1 (6–25% positive), 2 (26–50% positive), 3 (51–75% positive), or 4 (>75% positive). Staining intensity was scored on a scale of 0–3 as follows: 0, negative; 1, buff; 2, yellow; and 3, brown. The percentage of positive cells and the staining intensities were then multiplied to generate the immunoreactivity score for each case. Overall staining scores from 0 to 2, 3–6 and ⩾7 were considered negative, weak and strong expression, respectively. Both weak and strong expressions were considered positive.

### Statistical analyses

Data were expressed as mean±standard deviation. The prognostic significance of the expression of various markers was analyzed using the Kaplan–Meier method. The correlation between STAT1*β* and other clinical parameters was evaluated using a chi-square or Student’s *t*-test. Differences among the treatment groups were assessed using ANOVA and the appropriate statistical software (SPSS, IBM, USA). A *P*-value of ⩽0.05 was considered as statistically significant.

## Publisher’s Note:

Springer Nature remains neutral with regard to jurisdictional claims in published maps and institutional affiliations.

## Figures and Tables

**Table 1:** Correlation of STAT1 and STAT1*β* in 131 ESCC patient samples

	**STAT1 expression**		
**STAT1*****β*** **expression**	**Negative/Weak**	**Strong**	**Result**
Negative/Weak	67	17	*R=0.765
Strong	0	47	

*P<0.05

**Table 2:** Correlation of STAT1*β* expression with ESCC clinical parameters

	**Case number**	**STAT1*****β*** **expression**		
**Parameter**		**Negative/Weak**	**Strong**	**Result**
*Age*				
⩽57	94	62	31	*P*=0.356
>58	107	79	29	
				
*Gender*				
Male	150	100	50	*P*=0.071
Female	51	41	10	
				
*Tumor site*				
Upper	15	8	7	*P*=0.190
Middle	177	128	49	
lower	9	5	4	
				
*Differentiation*				
Poor	20	15	5	*P*=0.610
Intermediate	105	68	37	
Well	76	48	28	
				
*Tumor size*				
>5 cm	72	55	17	*P*=0.198
<5 cm	129	86	43	
				
*Depth of invasion*				
T1–T2	45	21	24	*P*<0.001*
T3–T4	156	120	36	
				
*Lymph node metastasis*				
Yes	96	75	22	*P*=0.045*
No	105	67	38	
				
*Clinical stage*				
1	22	11	11	*P*=0.026*
2	76	53	23	
3	91	71	20	
4	12	6	6	

*P<0.05
