# Supplementary figures and images for: STAT1β enhances STAT1 function by protecting STAT1α from degradation in esophageal squamous cell carcinoma
Source: Cell Death Dis. 2017 Oct 5;8(10):e3077–. doi: 10.1038/cddis.2017.481 (PMC5682650; doi:10.1038/cddis.2017.481)

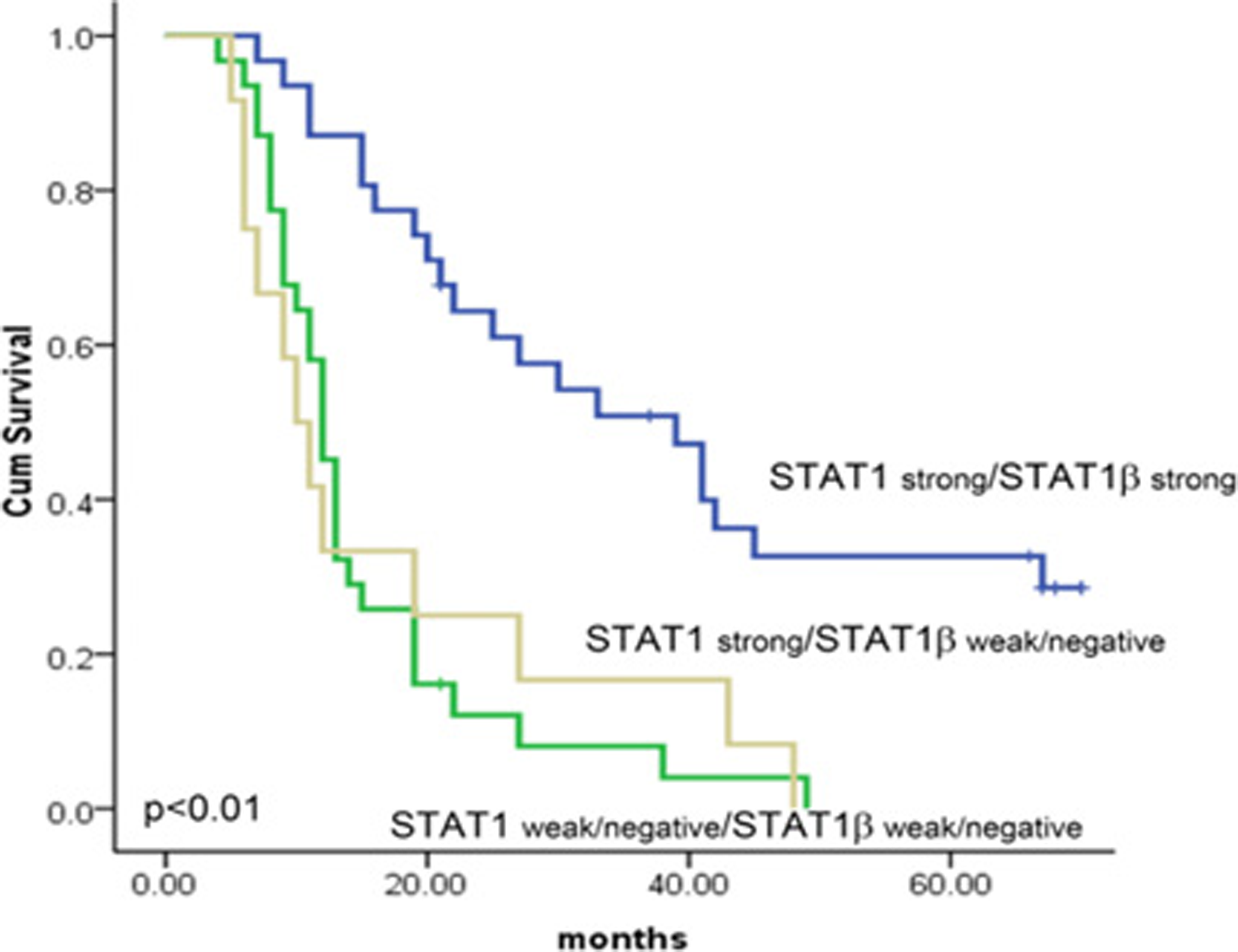

Supplement: Supplementary Figure [file cddis2017481x1.tif]
