# Supplementary material for: STAT1β enhances STAT1 function by protecting STAT1α from degradation in esophageal squamous cell carcinoma
Source: Cell Death Dis. 2017 Oct 5;8(10):e3077–. doi: 10.1038/cddis.2017.481 (PMC5682650; doi:10.1038/cddis.2017.481)
Supplement: Supplementary Figure Legend [file cddis2017481x2.docx]

**Supplemental Figure Legend**

**Supplemental Figure 1.** **Survival analysis of three subgroups of ESCC patients, based on STAT1 and STAT1β expression.** Kaplan-Meier analysis shows significant correlation between overall survival and the expression level of STAT1β when patient samples were divided into STAT1-strong/STAT1β-strong, STAT1-strong/STAT1β-weak/negative and STAT1-weak/negative/STAT1β-weak/negative subgroups (*p*<0.01).
